# The effect of an elective cesarean section on maternal request on peripartum anxiety and depression in women with childbirth fear: a systematic review

**DOI:** 10.1186/s12884-017-1371-z

**Published:** 2017-06-19

**Authors:** Renske M. Olieman, Femke Siemonsma, Margaux A. Bartens, Susan Garthus-Niegel, Fedde Scheele, Adriaan Honig

**Affiliations:** 1 Department of Psychiatry, VU University Medical Center, Amsterdam, The Netherlands; 2 Department of Obstetrics and Gynaecology, OLVG Hospital Location West, Amsterdam, The Netherlands; 3 Department of Psychiatry, OLVG Hospital Location West, Amsterdam, The Netherlands; 4 Department of Child Health, Norwegian Institute of Public Health, Oslo, Norway; 5 Institute and Outpatient Clinics of Psychotherapy and Psychosomatic Medicine, University Hospital Carl Gustav Carus, Fetscherstraße 74, 01307 Dresden, Germany

**Keywords:** Elective cesarean section, Cesarean section on maternal request, Childbirth fear, Anxiety, Postpartum depression, Caesarean

## Abstract

**Background:**

Obstetricians are often reluctant to grant requests for an elective cesarean section (ECS) due to childbirth fear. To date, it is unknown if an ECS on request improves mental well-being in the mother in the peripartum period and if possible beneficial effects on anxiety and depression could outweigh the increased risk of complications associated with a surgical delivery. A systematic review was conducted to explore the effect of ECS on request on peripartum anxiety and depression.

**Methods:**

We searched on PubMed, PsychoInfo and Embase. Studies were included with primary data on anxiety and/or depression postpartum in women with childbirth fear who had requested an ECS. After full-text evaluation of 65 papers and quality analysis of four papers, three papers were included. Of one paper additional and yet unpublished data were provided. Studies varied in outcome measures, hence no meta-analysis was performed.

**Results:**

Women who requested an ECS had higher antepartum depression and anxiety levels but no different postpartum depression levels than women who delivered vaginally. One study of good quality examined the effect of vaginal delivery in women preferring ECS: These women had significantly higher symptom levels of post-traumatic stress disorder (PTSD) and depression than women with normal vaginal delivery.

**Conclusions:**

The prospect of an ECS does not lower antepartum anxiety and/or depression levels in women requesting an ECS. If women resolutely persist in wishing an ECS despite adequate counselling and/or psychiatric treatment, the risk of developing depressive and PTSD symptoms in case of vaginal delivery should be taken into account, and an ECS may be considered as a valid alternative.

**Electronic supplementary material:**

The online version of this article (doi:10.1186/s12884-017-1371-z) contains supplementary material, which is available to authorized users.

## Background

In the past few decades, cesarean section rates have steadily increased worldwide, and in many places they exceed 20% of all deliveries [1–10]. Although cesarean sections can prevent maternal and perinatal mortality and morbidity, they are also associated with short-term and long-term risks which can extend many years beyond the current delivery and affect the health of the woman and her child as well as future pregnancies [11–17]. Besides health risks, a cesarean birth is also associated with higher costs [3, 11].

The rising numbers are partly due to the rising number of women requesting an elective cesarean section (ECS) [12, 18–20]. ECS on maternal request only are internationally estimated at 1 to 9% of all cesarean deliveries [17, 18, 21–23]. Often this request is due to psychosocial reasons [12, 18–20] and more specifically to fear of childbirth, which is experienced by approximately 20% of all pregnant women, with 6% to 10% experiencing severe childbirth fear [12, 18, 24]. These women are not only more likely to request a cesarean section [25–27] but are also more likely to receive an ECS [21, 28, 29]. Often, they are known with a psychiatric disorder and/or symptomatology [18, 30].

Because of the increased obstetric risks associated with cesarean sections, obstetricians are often reluctant to grant the wish for an ECS. On the other hand, obstetricians’ insistence on a vaginal delivery could induce additional stress and possibly increase the risk for peripartum anxiety and depression. To date, it is unknown if the prospect of an ECS on request improves mental well-being in the mother in the peripartum period and if possible beneficial effects on anxiety and depression could outweigh the increased risk of complications associated with a surgical delivery [16].

While other articles and systematic reviews concentrated mostly on the exploration of the effect of psychological or supportive interventions during pregnancy in women with childbirth fear [31–33], this systematic review was conducted to explore the impact of the prospect of an ECS itself as a possible intervention in the treatment of anxiety and depressive disorders during pregnancy. The control group consisted of women without a wish for ECS with a normal vaginal delivery.

We formulated the following research questions:

1. What is the effect of an ECS on maternal request on postpartum anxiety and depression levels in women with childbirth fear?

2. How do depression and anxiety levels develop peripartum in women with childbirth fear with an ECS on maternal request?

## Methods

PRISMA-guidelines [34] were followed. The research protocol was registered at PROSPERO (registration number: CRD42016041342).

### Search strategy and data sources

A PubMed, Embase and PsycInfo search was conducted on April 6th 2017. Search strategies were developed for each database in collaboration with a medical information specialist. Studies were included if they were published in English, French, German or Dutch and if they reported original data on anxiety and/or depression during and/or after pregnancy in women who received an ECS. The following set of keywords was used: (cesarean section (mesh) or abdominal delivery or postcesarean) AND ((anxiety (mesh) or panic or fear) or (depression (mesh) or dysthymia or melancholia)). The complete search strategy is shown in Additional file 1.

### Study selection

**Fig. 1:**
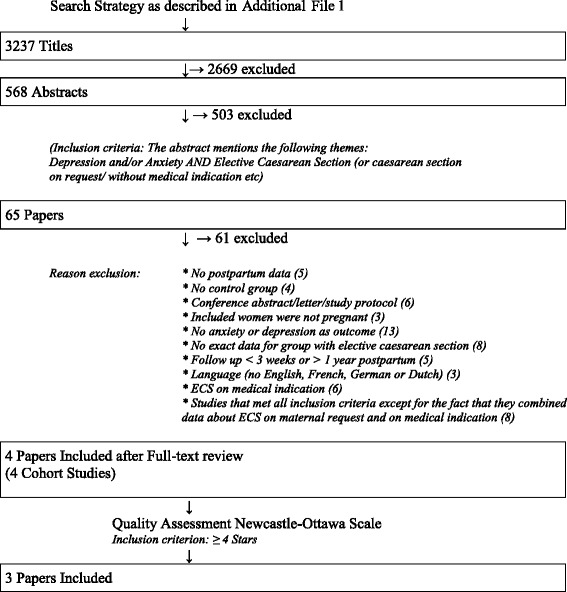
Flowchart

Two reviewers independently assessed inclusion eligibility. Initial selection for inclusion was based on screening of titles. Thereafter, included titles were screened on abstract. Abstracts were included if they mentioned depression and/or anxiety AND ECS/cesarean section on request. Following this, full-text versions of the selected studies were assessed for eligibility. Disagreement about inclusion was solved through discussion. Exclusion criteria are shown in Fig. 1. Studies were only included if they used validated screening tools, and studies would be compared by differences in mean scores. We tried to contact the first author if part of the needed data was missing.

### Quality assessment

To consider if the quality of the included studies was good enough, quality assessment was conducted utilizing the Newcastle-Ottawa quality assessment Scale [NOS, Additional file 2]. This is an assessment scale recommended by the Cochrane Collaboration [35]. The reviewers resolved differences in quality assessment through consensus. The comparability category included evaluation whether studies had been controlled for confounders influencing the primary outcome, i.e. risk factors for anxiety and depression postpartum. These risk factors were psychological factors (anxiety or depression in history or antepartum), personality traits (neuroticism), parity, psychosocial factors, age of the mother, level of education, and somatic complications. Antepartum depression and/or anxiety was considered to be the most important risk factor [16, 36–44].

## Results

### Description of inclusion and exclusion

A flowchart of the study selection is shown in Fig. 1. After full-text review (*n* = 65), 61 papers were excluded. Main reasons for paper exclusion were the absence of anxiety or depression data as an outcome (*n* = 13), no data available of the ECS-group (*n* = 8) and combining data of ECS on maternal request and on medical indication (*n* = 8). One study [45] was excluded because of low quality (Newcastle-Ottawa Assessment Scale: three stars). Three cohort studies, all published in English, remained. Table 1 shows a description of the three included studies. The studies varied in outcome measures, which precluded a meta-analysis. A narrative synthesis (qualitative appraisal) of the findings was conducted.

**Table 1:** Overview of the Included Studies

First Author (Year) Country	N	Design	Anxiety and/or depression scale	Follow-up	Results	Quality Appraisal (Newcastle Ottawa Scale)
Adams [16] (2011) Norway	(wish for) ECS = 5,352 VAG = 50,462	Cohort	SCL-8	30wk preg 6 mnth PP	Anxiety and depression scores ECS > VAG (AP and PP) ^*^. Difference PP is not significant anymore when adjusted for SCL-8 score AP.	8 stars (very good) Selection ++++ Comparability ++ Outcome ++
Garthus-Niegel [36] (2014) Norway	Prefer ECS, get VAG = 112 Prefer ECS, get ECS = 53 Normal VAG = 1,493	Cohort	WDEQ SCL-Anxiety IES EPDS	17wk preg 32wk preg 8wk PP	Women who prefer ECS but deliver vaginally have; Higher depression and anxiety scores AP *** Higher post-traumatic stress PP ^***^ and PPD ** compared to women without preference for ECS who deliver vaginally	7 stars (good) Selection ++++ Comparability ++ Outcome +
Wiklund [14] (2007) Sweden	(wish for) ECS = 91 VAG = 266	Cohort	EPDS	2 days PP 3mnth PP	PPD ECS = VAG	4 stars (satisfactory) Selection +++ Comparability - Outcome +

*AP* antepartum, *ECS* elective cesarean section, *EPDS* Edinburgh Postnatal Depression Scale, *IES* Impact of Event Scale (measures PTSD), *mnth* months, *preg* pregnancy, *PP* postpartum, *PPD* postpartum depression scores, *SCL-Anxiety* (Hopkins) Symptom-Checklist (measures anxiety), *SCL-8* (Hopkins) Symptom-Checklist-8 (measures anxiety and depression), *VAG* vaginal delivery, *wk* weeks, *WDEQ* Wijma Delivery Expectancy/Experience Questionnaire (measures childbirth fear)

* = p ≤ 0.05

** = *p* ≤ 0.01

*** = *p* ≤ 0.001

Table 1 shows the outcome of the quality appraisal by means of the Newcastle-Ottawa Assessment Scale. One study was considered to be of very good quality (8–9*) [16], the second study was considered to be of good quality (6–7*) [36], and the third study was considered to be of satisfactory quality (5–4*) [14]. Selection criteria of all studies were adequate, with clear cohort selection and good representativeness.

### Measurement of the preference for an ECS

All three included studies presented postpartum data of women with an elective cesarean section on maternal request. Adams et al. [16] and Garthus-Niegel et al. [36] based the preference for a cesarean delivery on the following question around 30–32 weeks of gestation: ‘If I could choose, I would (rather) have a cesarean delivery’. Wiklund et al. [14] included women that requested an ECS and the reason for this request as stated in their medical record.

### Anxiety and depression outcomes

**Fig. 2:**
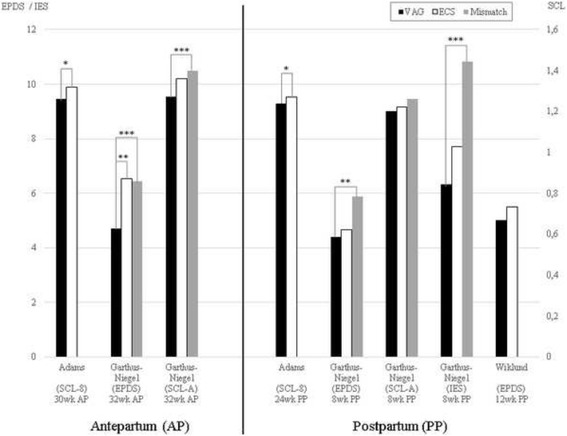
Mean Scores Anxiety and/or Depression Scale. * = p ≤ 0.05, ** = *p* ≤ 0.01, *** = *p* ≤ 0.001. AP = antepartum, ECS = elective cesarean section, EPDS = Edinburgh Postnatal Depression Scale, IES = Impact of Event Scale (measures PTSD), Mismatch = prefer ECS, deliver vaginally, PP = postpartum, SCL-Anxiety = (Hopkins) Symptom-Checklist (measures anxiety), SCL-8 = (Hopkins) Symptom-Checklist-8 (measures anxiety and depression), VAG = normal vaginal delivery, wk = weeks

The main outcomes of mean anxiety and depression scores are shown in Fig. 2. Adams et al. [16] and Garthus-Niegel et al. [36] found higher depression scores antepartum in women who received ECS on request than in women with vaginal delivery. Both studies adjusted for most of the risk factors, including parity. The study of Adams et al. [16] shows that the score on the Hopkins Symptom Checklist (SCL-8, a scale that measures both depression and anxiety) [46] was a significantly higher ante- and postpartum for women who received an ECS than for women who delivered vaginally. However, the differences in postpartum depression and anxiety scores were no longer significant when adjusted for antepartum data. In their sample, 5,352 women had a wish for ECS. They repeated the analyses in these women and in the 50,462 women with no such wish, finding no association of ECS with decline in SCL-8 score in either group.

Garthus-Niegel et al. [36] focused specifically on the preference for mode of delivery. They compared women with and without preference for ECS. Women who preferred an ECS but delivered vaginally were labelled as a ‘mismatch group’. These women showed higher levels of childbirth fear (Wijma Delivery Expectancy/Experience Questionnaire (WDEQ)) [47] score 72.02 versus 54.66^***^), higher scores of antepartum anxiety (SCL total score 13.93 versus 12.66^***^) and higher scores of PTSD two months postpartum (Impact of Event Scale (IES)) [48] 10.83 versus 6.32^***^) than women without this preference who delivered vaginally. Garthus-Niegel et al. [36] only published antepartum data on anxiety and depression. On request, however, S. Garthus-Niegel provided us with postpartum data that have not been published yet. Besides higher antepartum depression scores (Edinburgh Postnatal Depression Scale (EPDS)) [49, 50] 6.44 versus 4.72 antepartum^***^), the unpublished postpartum data showed that the mismatch group also had higher depression scores postpartum (EPDS [49, 50] 5.87 versus 4.44 postpartum^**^) than women without a preference for ECS who delivered vaginally. In contrast, women whose request for an ECS was granted (the match group) still had higher antepartum depression scores (EPDS 6.54 versus 4.72^**^ in the group without preference for ECS), but their postpartum depression scores normalized (4.58 (match group) versus 4.44 (normal vaginal delivery group) versus 5.87 (mismatch group)). The postpartum anxiety levels normalized for both the match group and the mismatch group (SCL total score 12.25 (match group) versus 12.03 (normal vaginal delivery group) versus 12.58 (mismatch group)).

In the third included study that specifically focused on ECS on maternal request, Wiklund et al. [14] showed that first-time mothers who received an ECS on request had the same

depression (EPDS) scores three months postpartum as women who delivered vaginally. This in line with the postpartum data of Garthus-Niegel et al. and the adjusted postpartum data of Adams et al.. The study of Wiklund et al. also showed that women requesting an ECS had a better birth experience if this request was met, compared to women who had planned a vaginal delivery after requesting an ECS.

## Discussion

The focus of this review was the effect of an ECS on maternal request on the mother’s peripartum anxiety and depression levels in women with childbirth fear. Antepartum depression and anxiety scores remained high in women requesting and receiving an ECS, but postpartum depression scores were the same as in women without a wish for ECS who delivered vaginally. These findings are supported by eight studies [9, 12, 43, 44, 51–54] of adequate quality that combined data of ECS on medical indication and on maternal request (data and quality assessment not shown). One study of 112 women with a wish for ECS but who delivered vaginally, reported higher scores for depression and PTSD after childbirth [36].

The fact that antepartum scores of anxiety or depression remain high in women with childbirth fear despite the prospect of an ECS are in line with other studies measuring antepartum anxiety levels in women undergoing an ECS [18, 19, 55, 56].

Because none of the studies had data prior to conception, we cannot conclude whether depressive women more often request an ECS or if childbirth fear itself leads to depressive symptoms. The study of Størksen et al. [18] concluded that women who requested an ECS had several vulnerability characteristics, such as poor mental health, previous negative overall birth experiences and poor social support. A mismatch in wish and actual delivery mode seems to be an extra stressor for these vulnerable women. The only included study that explicitly focused on ‘mismatches’ is the study of Garthus-Niegel et al. [36]. Their results show that granting the maternal request for an ECS may lower the risk of developing PTSD and postpartum depression in these women. Comparison of the ECS match group (wish for ECS granted) and the mismatch group (preference for ECS, but not performed) showed a trend of higher PTSD symptoms postpartum in the mismatch group (IES is 7.7 in match group versus 10.83 in mismatch group (*p* = 0.018 and 0.11 after post hoc Bonferroni tests)). There also was a trend towards higher postpartum depression levels in the mismatch group (EPDS is 4.58 in the match group versus 5.87 in the mismatch group), but these differences were not statistically significant (*p* = 0.066, no post hoc tests were applied)). This may have been due to a lack of power, as the match group consisted of only 53 women and the mismatch group consisted of 112 women (versus 1493 women in the group of normal vaginal delivery, whose PTSD and depression levels were significantly lower than those of the mismatch group but similar to those of the match group). Also, post hoc Bonferroni tests were used.

Another mismatch that could be evaluated are women who prefer a vaginal delivery but deliver through cesarean section. The study of Houston et al. [57] showed that a stronger preference for vaginal delivery was associated with higher depression scores postpartum among women who underwent cesarean section, indicating that it may not be the delivery method itself that induces stress, but the mismatch in expectations.

The question that remains is the meaning of these findings for clinical practice, given the present limited data. Emphasizing the risk of a cesarean section does not seem to be sufficient to persuade all women to deliver vaginally if this is medically viable. Rather, fear of childbirth and a maternal request for ECS should be taken seriously and should be further explored. In daily practice, routine screening of women who request an ECS should be considered, for example with a questionnaire like the SCL-8 [46]. If a woman scores above cut-off, further evaluation by a psychiatrist or psychologist is needed, as research has shown that self-reported screening tools for perinatal depression yield a higher rate of positive cases than clinical interview methods [41]. However, if a woman resolutely persists in her wish for ECS despite counselling and/or psychiatric treatment, the risk of developing depressive and PTSD symptoms should be taken into account, and consequently an ECS may be considered as a valid option.

The best psychiatric treatment of childbirth fear is still under debate. Several options have been described, and some studies have shown that at least half of the women can prepare for a normal vaginal delivery and that the rate of vaginal deliveries increased after treatment by group psychoeducation combined with relaxation exercises [31, 32]. Other studies that evaluated treatment of childbirth fear by supportive, psychotherapeutic or cognitive treatment did not show a decrease in ECS rates [24, 58] nor a more positive experience of delivery [33]. A comprehensive systematic review of Weaver et al. [59] on the impact of planned interventions offered to women requesting an ECS concluded that more research is needed to identify how tokophobic women might best be helped.

There are several strengths and limitations to this review. To the best of our knowledge, this is the first systematic review on the effect of an ECS on maternal request on peripartum anxiety and depression. A qualitative appraisal of the studies made it possible to highlight the studies with the best quality. We did not include the studies that combined data of ECS on medical indication and on maternal request. More research is needed specifically concerning ECS on maternal request, including the focus on mismatches between the maternal wish and actual mode of delivery. It is important that these studies contain information on the mental health of pregnant women prior to delivery, to assure that possible differences in mental health postpartum may not just reflect pre-existing differences between the groups. Data about the motivation for requesting an ECS should be included, as should data about adequate counselling about the risks of surgical delivery. Clarification of obstetric factors would further strengthen such research.

## Conclusions

Women who requested an ECS had higher antepartum depression and anxiety levels than women who had planned to deliver vaginally. If the request for ECS was granted, their antepartum depression and anxiety levels did not decline, but postpartum depression levels reverted to normal. One study of good quality reported that, if ECS was not granted, women who persisted in preferring ECS had significantly higher symptom levels of post-traumatic stress disorder (PTSD) and depression after vaginal delivery than women who had planned on vaginal delivery. For clinical practice, given these limited data, this means that this vulnerable group of women need adequate counselling and psychiatric treatment for possible anxiety and/or depressive disorder. However, if women resolutely persist in their wish for a cesarean section, an ECS may be considered as a valid alternative.

## Additional files

Additional file 1: Search strategy (April 6th 2017). (DOC 24 kb)

Additional file 2: Newcastle- Ottawa Quality Assessment Scale. http://www.ohri.ca/programs/clinical_epidemiology/oxford.asp. (DOC 37 kb)

## Abbreviations

ECS: Elective cesarean section
EPDS: Edinburgh postnatal depression scale
IES: Impact of event scale
PTSD: Post traumatic stress disorder
SCL-8: (Hopkins) Symptom-Checklist-8
SCL-Anxiety: (Hopkins) symptom-checklist
WDEQ: Wijma delivery expectancy/experience questionnaire
