# Supplementary material for: The effect of an elective cesarean section on maternal request on peripartum anxiety and depression in women with childbirth fear: a systematic review
Source: BMC Pregnancy Childbirth. 2017 Jun 19;17:195. doi: 10.1186/s12884-017-1371-z (PMC5477251; doi:10.1186/s12884-017-1371-z)
Supplement: Supplementary file 1 — Search strategy (April 6th 2017). (DOC 24 kb) [file 12884_2017_1371_MOESM1_ESM.doc]

**Appendix S1**

**Pubmed**

((postcesarean*[tiab] OR postcaesarean*[tiab] OR "Cesarean Section"[Mesh] OR cesarean*[tiab] OR caesarean*[tiab] OR c-section*[tiab] OR abdominal deliver*[tiab] OR caesarian*[tiab] OR cesarian*[tiab] OR postcaesarian*[tiab] OR postcesarian*[tiab]) AND (("Panic"[Mesh] OR Anxiety[tiab] OR “Anxiety”[Mesh] or “Anxiety Disorders”[Mesh] OR panic*[tiab] OR fear[tiab]) OR ("Depression"[Mesh] OR "Depressive Disorder"[Mesh] OR depress*[tiab] OR melanchol*[tiab] OR dysthym*[tiab]))

**Psychinfo**

( ( DE "Depression (Emotion)" OR DE "Major Depression" OR DE "Anaclitic Depression" OR DE "Dysthymic Disorder" OR DE "Endogenous Depression" OR DE "Postpartum Depression" OR DE "Reactive Depression" OR DE "Recurrent Depression" OR DE "Treatment Resistant Depression" OR DE "Atypical Depression") OR ( TI ( depress* OR melanchol* OR dysthym*) OR AB ( depress* OR melanchol*) OR dysthym* ) ) OR ( ( DE "Anxiety Disorders" OR DE "Acute Stress Disorder" OR DE "Castration Anxiety" OR DE "Death Anxiety" OR DE "Generalized Anxiety Disorder" OR DE "Obsessive Compulsive Disorder" OR DE "Panic Disorder" OR DE "Phobias" OR DE "Posttraumatic Stress Disorder" OR DE "Separation Anxiety" OR DE "Anxiety" OR DE "Computer Anxiety" OR DE "Mathematics Anxiety" OR DE "Performance Anxiety" OR DE "Social Anxiety" OR DE "Speech Anxiety" OR DE "Test Anxiety" OR DE "Panic Attack" OR DE "Social Anxiety" ) OR ( TI ( anxiety OR panic* OR fear ) OR AB (anxiety OR panic* OR fear) ) ) AND TI (Postcesarean* OR postcaesarean* OR cesarean* OR caesarean* OR c-section* OR abdominal deliver* OR caesarian* OR cesarian* OR postcaesarian* OR postcesarian* OR AB Postcesarean* OR postcaesarean* OR cesarean* OR caesarean* OR c-section* OR abdominal deliver* OR caesarian* OR cesarian*OR postcaesarian* OR postcesarian*)

**Embase**

('anxiety'/exp OR 'anxiety disorder'/exp OR 'depression'/exp OR anxiety:ab,ti OR panic*:ab,ti OR depress*:ab,ti OR melanchol*:ab,ti OR dysthym*:ab,ti OR fear:ab,ti) AND (('cesarean section'/exp) OR (postcesarean:ab,ti OR postcaesarean:ab,ti OR cesarean*:ab,ti OR caesarean*:ab,ti OR caesarian*:ab,ti OR cesarian*:ab,ti OR postcaesarian*:ab,ti OR postcesarian*:ab,ti) OR (c NEXT/1 section*:ab,ti) OR (abdominal NEXT/1 deliver*:ab,ti))
