# Supplementary material for: The effect of an elective cesarean section on maternal request on peripartum anxiety and depression in women with childbirth fear: a systematic review
Source: BMC Pregnancy Childbirth. 2017 Jun 19;17:195. doi: 10.1186/s12884-017-1371-z (PMC5477251; doi:10.1186/s12884-017-1371-z)
Supplement: Supplementary file 2 — Newcastle- Ottawa Quality Assessment Scale. http://www.ohri.ca/programs/clinical_epidemiology/oxford.asp. (DOC 37 kb) [file 12884_2017_1371_MOESM2_ESM.doc]

**Appendix S2**

**COHORT STUDIES**

*Selection* (Maximum 4 stars)

1) Representativeness of the exposed cohort

1. Truly representative of the average woman in the community **✵**
2. Somewhat representative of the average woman in the community **✵**

c) Selected group of users, e.g. nurses, volunteers

d) No description of the derivation of the cohort

2) Selection of the non-exposed cohort

1. drawn from the same community as the exposed cohort **✵**
2. drawn from a different source

c) no description of the derivation of the non-exposed cohort

3) Ascertainment of exposure

1. secure record (e.g. surgical records) **✵**
2. structured interview **✵**

c) written self-report

d) no description

4) Demonstration that outcome of interest was not present at start of study

a) yes **✵**

b) no

*Comparability* (Maximum 2 stars)

1) Comparability of cohorts on the basis of the design or analysis

a) study controls for antepartum depression and anxiety (select the most important factor) **✵**

b) study controls for any additional factor **✵**

*Outcome* (Maximum 3 stars)

1) Assessment of outcome

1. independent blind assessment **✵**
2. record linkage **✵**

c) self-report

d) no description

2) Was follow-up long enough for outcomes to occur

1. yes
2. no

3) Adequacy of follow up of cohorts

a) complete follow up - all subjects accounted for **✵**

b) subjects lost to follow up unlikely to introduce bias (>80 % follow up, or description provided of those lost) **✵**

c) follow up rate < 60% or follow-up <80% and >60% without description

d) no statement

8-9 stars= very good quality

6-7 stars= good quality

4-5 stars= satisfactory quality

0-3 stars= unsatisfactory quality
